# Supplementary material for: Optimizing TMS Coil Placement Approaches for Targeting the Dorsolateral Prefrontal Cortex in Depressed Adolescents: An Electric Field Modeling Study
Source: Biomedicines. 2023 Aug 21;11(8):2320. doi: 10.3390/biomedicines11082320 (PMC10452519; doi:10.3390/biomedicines11082320)
Supplement: Supplementary file 1 [file biomedicines-11-02320-s001.zip › biomedicines-2478993-supplementary.pdf]

Supplementary Materials:

S1. Coordinate transformation

For the pilot clinical trial, coil targets were marked using the Medtronic StealthStation™ S7 equipped with an AxiEM frameless localization system running Synergy Cranial 2.2.6. The surgical navigation software has a proprietary coordinate system that needed to be transformed into MRI coordinates.

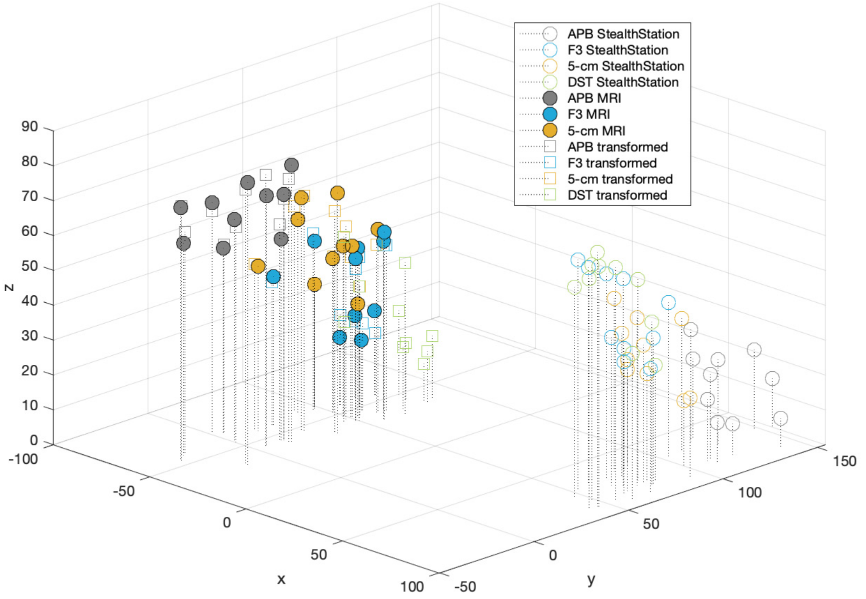

Figure S1. Individual scalp targets in StealthStation coordinates (empty circles), MRI fiducial markers (filled circles), and the transformed target coordinates in MRI space (squares).

We first performed 3D rendering the individual MRIs in MATLAB and manually marked the center of the fiducials (APB, 5-cm, and F3 targets) on the scalp. The ensemble of target coordinates from all ten subjects were used to derive the affine transformation from the surgical navigation system to MRI space. The DLPFC Scalp Target (DST) for individual subjects were subsequently transformed to MRI space for the E-field simulations. Figure S1 shows the individual scalp targets in StealthStation coordinates (circles), MRI fiducial markers (stars), and the StealthStation-to-MRI transformed coordinates (squares). The error between the manual MRI fiducial markers and the final transformed target coordinates is less than 2 mm in the x and y directions, and less than 6 mm in the z direction.
